# Identification of genes regulating ovary differentiation after pollination in hazel by comparative transcriptome analysis

**DOI:** 10.1186/s12870-018-1296-3

**Published:** 2018-05-09

**Authors:** Yunqing Cheng, Yuchu Zhang, Chunming Liu, Pengfei Ai, Jianfeng Liu

**Affiliations:** 1 Jilin Provincial Key Laboratory of Plant Resource Science and Green Production, Jilin Normal University, Siping, 136000 Jilin Province China; 2 College of Bioscience & Bioengineering, Hebei University of Science and Technology, Shijiazhuang, 050080 Hebei Province China

**Keywords:** Hazel, Ovary, Ovule, Transcriptome

## Abstract

**Background:**

Hazel (*Corylus* spp.) exhibits ovary differentiation and development that is initiated from the ovary primordium after pollination, conferring the plant with a unique delayed fertilization. Failure of development of the ovary and ovule after pollination can lead to ovary abortion and blank fruit formation, respectively, with consequent yield loss. However, the genes involved in ovary and ovule differentiation and development are largely unknown.

**Results:**

In unpollinated pistillate inflorescences (stage F), the stigma shows an extension growth pattern. After pollination, a rudimentary ovary begins to form (stage S), followed by ovule differentiation (stage T) and growth (stage FO). Total RNA was obtained from pistillate inflorescences or young ovaries at stage F, S, T and FO, and sequencing was carried out on a HiSeq 4000 system. De novo assembly of sequencing data yielded 62.58 Gb of nucleotides and 90,726 unigenes; 5524, 3468, and 8714 differentially expressed transcripts were identified in F-vs-S, S-vs-T, and T-vs-FO paired comparisons, respectively. An analysis of F-vs-S, S-vs-T, and T-vs-FO paired comparisons based on annotations in the Kyoto Encyclopedia of Genes and Genomes revealed six pathways that were significantly enriched during ovary differentiation, including ko04075 (Plant hormone signal transduction). Auxin level increased after pollination, and an immunohistochemical analysis indicated that auxin was enriched at the growth center of pistillate inflorescences and young ovaries. These results indicate that genes related to auxin biosynthesis, transport, signaling, the floral quartet model, and flower development may regulate ovary and ovule differentiation and development in hazel.

**Conclusions:**

Our findings provide insight into the molecular mechanisms of ovary differentiation and development after pollination in this economically valuable plant.

**Electronic supplementary material:**

The online version of this article (10.1186/s12870-018-1296-3) contains supplementary material, which is available to authorized users.

## Background

Hazel (*Corylus* spp.) is the most economically important plant of the Betulaceae family. Turkey and Italy produce more than 80% of the world’s hazelnut crop. Other important hazelnut producers are the U.S. (3.8%), Azerbaijan (3.3%), Spain (2.6%), Iran (1.9%), Georgia (1.9%), and China (1.7%) [1]. In the last decade, the area devoted to the culture of hybrid hazel (*C. heterophylla* × *C. avellana*) has increased dramatically in China and is currently more than 50,000 hm^2^ with a concomitant increase in fruit yield, setting the stage for China to become a major global producer of hazelnuts. Northeast China is the largest production area, with the hazelnut industry significantly impacting regional economic development.

In angiosperms, stigma growth, pollination, fertilization, and subsequent ovary and ovule development in pistillate inflorescences are the requirements for fruit formation, which determines plant yield. The mature gynoecium has four main components: stigma, style, ovary, and gynophore [2]. In general, the mature gynoecium at anthesis is ready for fertilization [3]. Compared to *Arabidopsis*, hazel has distinct pistillate inflorescences and a different mode of fruit development: at anthesis of pistillate inflorescences, the gynoecium is still immature due to the absence of a complete ovary and ovule, and the stigma and style form a complex [4]; at the bottom of this complex are a few layers of ovary primordium cells [5, 6]. After pollination, pollen tubes reach to the bottom of the style, which is also known as the pollen tube cavity. The early ovary and ovule primordium then begin to differentiate. After mature embryo sacs are formed, pollen tubes regrow and enter the ovary, and complete fertilization about 2 months after pollination [6]. Pollen tube growth signaling has an important impact on ovary and ovule differentiation in hazel. Ovary and ovule abortion are common during pistillate inflorescence and fruit development [7, 8]. Ovary abortion is partly caused by the absence of a mature embryo sac, and determines the number of fruit per cluster [5]. A complete embryo structure can be observed in an abortive ovule, which is closely associated with blank fruit formation [8]. Most of the genes that regulate ovary and ovule development in hazel are not known. Given the unique pistillate inflorescence and fruit development characteristics of this plant, identifying these genes will provide insights into the mechanism of ovary development and yield loss caused by frequent ovary and ovule abortion during the fruit development stage.

In the present study, we analyzed gene expression in pistillate inflorescences and young fruits during four developmental stages by Illumina HiSeq 4000 analysis. We identified differentially expressed genes (DEGs) that may be involved in the regulation of ovary and ovule formation in hazel. Auxin was detected in pistillate inflorescences and young fruits by immunohistochemistry and HPLC-MS/MS (high-performance liquid chromatography-tandem mass spectrometry) in order to clarify its function in ovary development. Comparison of gene expression patterns at the four developmental stages of the ovary provides important insights into the molecular mechanisms of ovary differentiation and growth after pollination in hazelnut.

## Methods

### Plant material and treatment

The field experiment was conducted at a hazel orchard near Siping, Jilin Province, China. The experimental site is located in the North Temperate Zone and has a typical continental monsoon climate. Local hazel blooming and harvest dates are approximately April 20 and August 25, respectively.

In 2015, sixty 12-year-old hybrid hazel trees (*C. heterophylla* × *C. avellana*) cultivar ‘Dawei’, from Forest Research Institute of Liaoning Province, Dalian, China, were selected as study materials. On March 20 of that year, staminate shoots of the ‘PingOu 21’, ‘Yuzhui’, and ‘Liaozhen 7’ cultivars from Forest Research Institute of Liaoning Province were sampled for artificial pollination only and cultured in a light incubator; pollen was collected and the pollen germination ratio was determined as previously described [5]. After mixing in equal quantities, the pollen was stored at 4 °C until used for artificial pollination. On April 8, about 3000 pistillate inflorescences of ‘Dawei’ were randomly bagged and tagged. On April 20 (blooming date), about 1500 pistillate inflorescences were collected as unpollinated samples (stage F). Artificial pollination was carried out on the same day, and about 1000 pistillate inflorescences or young fruit clusters were sampled on May 10 when a rudimentary ovary began to develop (stage S). On May 30, about 120 pistillate inflorescences were sampled (early ovule formation, stage T); and on June 20, about 60 fruit clusters were sampled (achievement of fertilization and ovule growth, stage FO). After transport to the laboratory, the exterior bracts of pistillate inflorescences or fruit clusters were manually removed, and the stigma with the ovary primordium or ovary was isolated and collected, with three biological replicates prepared for each developmental stage. The samples were stored in liquid nitrogen until use. The voucher specimens of these materials have been publicly deposited in Shenyang Agriculture University, Shenyang, China. All field experiments were performed in accordance with the Convention on the Trade in Endangered Species of Wild Fauna and Flora.

### RNA extraction, library construction, and sequencing

In order to investigate changes in gene expression during the four developmental stages, 12 digital gene expression (DGE) profiling libraries were constructed: DWN-F-A (DWN: hybrid hazelnut cultivar ‘Dawei’), DWN-F-B, DWN-F-C, DWN-S-A, DWN-S-B, DWN-S-C, DWN-T-A, DWN-T-B, DWN-T-C, DWN-FO-A, DWN-FO-B, and DWN-FO-C. For the library names, ‘F’, ‘S’, ‘T’, and ‘FO’ indicate first, second, third, and fourth developmental stages and ‘A’, ‘B’, and ‘C’ indicate the three biological replicates. Total RNA was extracted from pistillate inflorescences or young ovaries using the RNA Easy spin Isolation System (Aidlab Biotech, Beijing, China). RNA pretreatment before sequencing was performed as previously described [9], and sequencing was carried out on a HiSeq 4000 system (Illumina, San Diego, CA, USA). Raw transcriptome data were deposited in the Sequence Read Archive (https://www.ncbi.nlm.nih.gov/SRA).

### Data analysis and mapping of DGE tags

After removal of reads with adaptors, reads with more than 5% unknown bases (N), and low quality reads (defined as reads in which the proportion of bases with a quality score < 10 was greater than 20%), the remaining clean reads were stored in FASTQ format. Trinity (version: v2.0.6) [10] was used to perform de novo assembly with clean reads from all 12 libraries with min_contig_length set to 150 and min_kmer_cov set to 3 and all other parameters set to default. Tgicl (version: v2.0.6) [11] was used to cluster transcripts to Unigenes with repeat_stringency, minmatch, and minscore set to 0.95, 35, and 35, respectively, and all other parameters set to default. We used Blast [12] to align unigenes to NT, NR, COG, KEGG and SwissProt to obtain annotations, Blast2GO (Version: v2.5.0; parameters: default) [13] with NR annotation to obtain GO annotations, and InterProScan5 [14] to obtain InterPro annotations. Clean reads were mapped to unigenes using Bowtie2 (version: v2.1.0) [15] with options “q; phred64; sensitive; dpad, 0; gbar, 99999999; mp, 1, 1; np, 1; score-min L, 0, -0.1; I, 1; X, 1000; no-mixed; no-discordant; p, 1; k, 200”, and then, gene expression levels were calculated with RSEM [16]; all parameters were set to default. For different sample libraries (F-vs-S, S-vs-T, and T-vs-FO, in which the former was used as the control and the latter as the experimental group in each paired comparison), three pairs of DGE profiles were compared in order to determine changes in gene expression during ovary differentiation and ovule growth in hazel. Based on the unigene expression results, DEGs were identified with EBseq [17] by setting the threshold of fold change as ≥2.00 and posterior probability of equivalent expression (PPEE) as ≤0.05. KEGG pathway enrichment analysis of DGE data was carried by a BLAST search of the KEGG database (http://www.kegg.jp/kegg/). Q ≤ 0.05 was used as the threshold for significant enrichment of DEG KEGG pathways. Hierarchical clustering analysis of DEGs was carried out using Multi Experiment Viewer (http://mev.tm4.org/#/welcome).

### Auxin localization and quantification

Fresh hazel ovary or stigma samples from four different developmental stages were fixed in 4% paraformaldehyde for 16 h at 4 °C, then washed with phosphate-buffered saline (PBS) and incubated in 2.0 M NaOH for 30–40 min at 65 °C. The softened samples were incubated in 0.1 M acetic acid for 30 s, rinsed three times with distilled water, boiled in 0.01 M sodium citrate buffer (pH 6.0) for antigen retrieval, and blocked in PBS with Tween and 1% bovine serum albumin. Anti-auxin antibody (1 mg/ml^− 1^) (Sigma-Aldrich, St. Louis, MO, USA) and fluorescein isothiocyanate- conjugated goat anti-mouse IgG (H + L) (Beyotime Institute of Biotechnology, Nanjing, China) were used as primary and secondary antibodies, respectively. Samples were mounted with anti-fade mounting medium (Beyotime Institute of Biotechnology) and cover slips, and imaged under a fluorescence microscope (Olympus, Tokyo, Japan) at an excitation wavelength of 494 nm.

The levels of auxin were determined by Zoonbio Biotechnology Co., Ltd. (Nanjing, China) using HPLC-MS/MS [18]. Auxin extraction and purification was performed as previously described using 0.5 g frozen samples. Then, the purified product was subjected to HPLC-MS/MS analysis. HPLC (Agilent 1260) analysis was carried out using a ZORBAX SB-C18 (Agilent Technologies) column (2.1 mm × 150 mm; 3.5 μm). The mobile phase A solvent consisted of water/0.1% methanoic acid, and the mobile phase B solvent consisted of ultrapure methanol. The injection volume was 10.0 μL. Parameters for MS analysis were set as follows: spray voltage, + 4500 V, −3000v; pressure of the air curtain, nebulizer, and aux gas were 15, 65, and 70 psi, respectively; atomizing temperature, 350 °C. Measurements were obtained for three biological replicates of each sample. Data were analyzed using SAS v.8.01 (SAS Institute, Cary, NC, USA) and significant differences between groups were evaluated with the least significant difference (LSD) test at a significance level of 5%.

### Quantitative real-time (qRT)-PCR analysis

The DGE results obtained by Illumina HiSeq 4000 sequencing were validated by qRT-PCR as previously described, using the same RNA samples [7]. Relative expression levels of selected genes were calculated with the 2^−ΔΔCt^ method [7]; primers used for qRT-PCR analysis are listed in Additional file 1: Table S1.

## Results

### Illumina sequencing and sequence assembly

To evaluate global changes in gene expression during ovary differentiation and ovule growth in hazel, 12 DGE libraries were sequenced with the Illumina HiSeq 4000 platform, generating approximately 52 million bases of raw reads for each library. The low-quality reads were filtered, yielding 625.79 million bases of clean reads. For all 12 libraries, de novo assembly of clean reads generated 62.58 Gb of nucleotides and 90,726 unigenes with a mean length of 1054 bp (Additional file 2: Table S2). All raw transcriptome data were deposited in the sequence read archive (accession no. SRS1980648). A query of unigenes against seven public nucleotide and protein databases yielded 67,946 (74.89%) annotated unigenes (Table 1).

**Table 1:** Summary of functional annotations

Values	Total	Nr Annotated	Nt Annotated	SwissProt Annotated	KEGG Annotated	COG Annotated	InterPro Annotated	GO Annotated	Overall
Number	90,726	60,659	60,865	42,927	37,636	26,110	47,356	11,596	67,946
Percentage	100%	66.86%	67.09%	47.31%	41.48%	28.78%	52.20%	12.78%	74.89%

NT, nucleotide sequence database, website: ftp://ftp.ncbi.nlm.nih.gov/blast/db; NR, non-redundant protein sequence database, website: ftp://ftp.ncbi.nlm.nih.gov/blast/db; GO, Gene Ontology, website: http://geneontology.org; COG: Cluster of Orthologous Groups of proteins, website: http://www.ncbi.nlm.nih.gov/COG; KEGG: Kyoto Encyclopedia of Genes and Genomes, website: http://www.genome.jp/kegg; SwissProt: UniProt Knowledgebase, website: http://ftp.ebi.ac.uk/pub/databases/swissprot; InterPro: InterPro database, website: http://www.ebi.ac.uk/interpro

### Global gene expression at four different developmental stages of ovaries

**Fig. 1:**
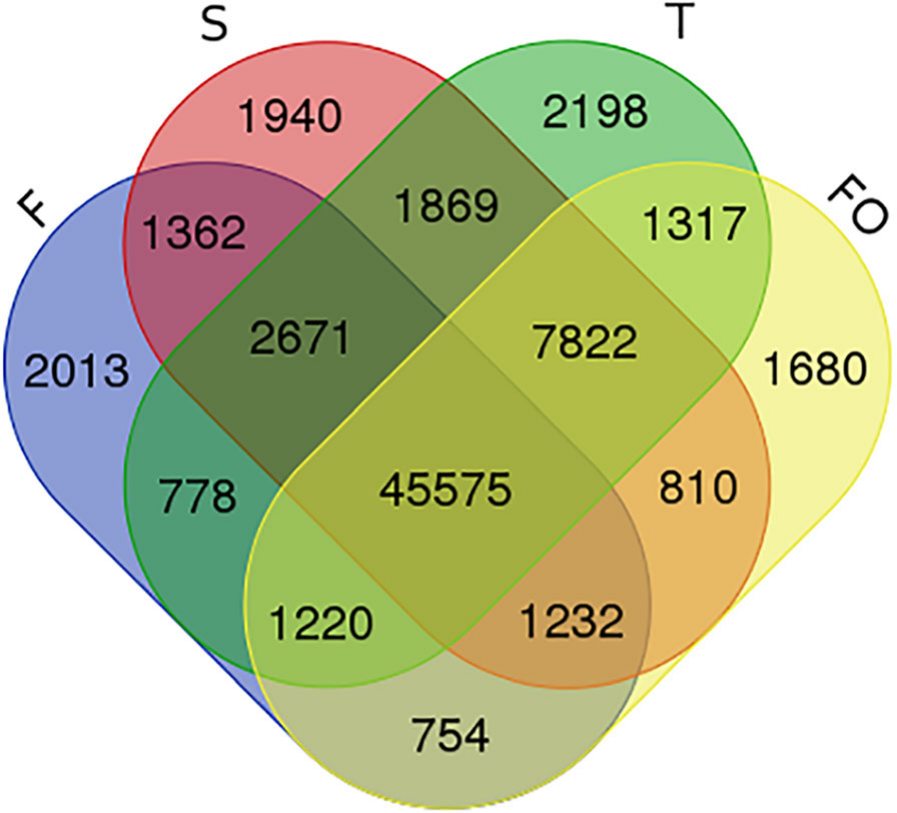
Venn diagram showing the overlaps of gene expression between the four different developmental stages in hybrid hazelnut

In total, 56,605, 63,281, 63,450, and 60,410 unigenes were recovered at stages F, S, T, and FO of ovary development, respectively (Fig. 1). The number of expressed genes was higher at stages S and T than at stages F and FO, indicating that more genes are needed to coordinate the initiation of the rudimentary ovary after pollination and ovule differentiation. Constitutive expression of 45,575 genes was found in all four developmental stages; 2013, 1940, 2198, and 1680 were expressed only at stages F, S, T, and FO, respectively. Genes with stage-specific expression are presumed to coordinate stigma development, rudimentary ovary development, ovule differentiation, and ovule growth. The ovule differentiation and ovule growth stages had the largest and smallest number of stage-specific genes, respectively.

### DEGs in paired comparisons of four different ovary development stages

**Fig. 2:**
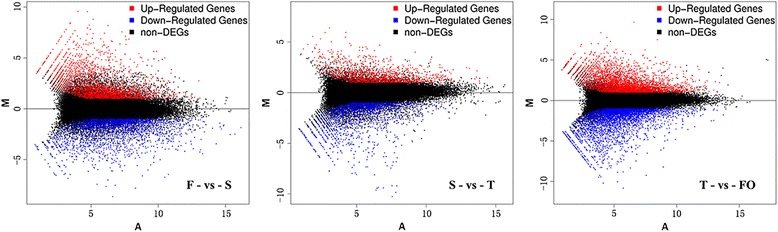
MA plot of DEGs. The MA-plot is a plot of the distribution of the red/green intensity ratio (‘M’) plotted by the average intensity (‘A’). M is the binary logarithm of the intensity ratio (or difference between log intensities) and A is the average log intensity for a dot in the plot. X axis represents value A (log2 transformed mean expression level), and Y axis represents value M (log2 transformed fold change). Red, blue and black points represent up-, down- and non-regulated DEGs respectively. “F” was the control and “S” was experimental group in “F-vs-S” paired comparison

A fold change threshold of ≥2.00 and a PPEE threshold of ≤0.05 were used to identify DEGs in the three paired comparisons (Fig. 2; Table 2). Similar numbers of expressed genes were found in the paired comparisons of F-vs-S (68,046), S-vs-T (68,794), and T-vs-FO (67,926) (Table 2). The number of DEGs in the F-vs-S paired comparisons was 5524 (2522 downregulated and 3002 upregulated; Additional file 3: Table S3); 3468 DEGs were found in the S-vs-T paired comparisons (1970 downregulated and 1498 upregulated; Additional file 4: Table S4); and 8714 DEGs were found in the T-vs-FO paired comparisons (4427 downregulated and 4287 upregulated; Additional file 5: Table S5). Thus, the highest number of DEGs was found in the T-vs-FO paired comparisons. The rudimentary ovary begins to form at stage S after pollination, and ovary and ovule sizes increase from stage T to FO. Our results indicate that many genes are activated upon pollination at stage S compared to stage F, as evidenced by the large number of upregulated DEGs, in order to meet the demands for material and energy during ovule growth at stage FO.

**Table 2:** Gene expression levels across different sample libraries

Differently expressed genes	F-vs-S	S-vs-T	T-vs-FO
Up-regulated	3002	1498	4287
Downregulated	2522	1970	4427
Total DEGs	5524	3468	8714
Not DEGs	62,522	65,326	59,212
Total expressed genes	68,046	68,794	67,926

### Analysis of DEG clustering and pathway enrichment

**Fig. 3:**
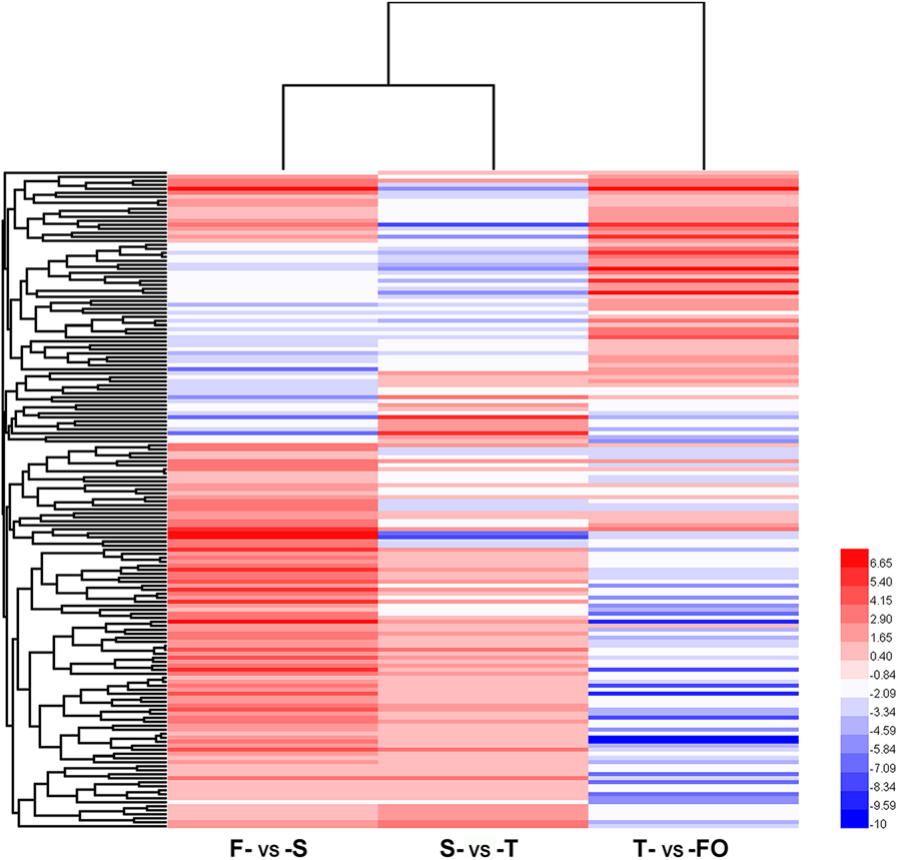
Hierarchical clustering (HCE) analysis of differentially expressed transcripts in F-vs-S, S-vs-T, and T-vs-FO paired comparisons. “F” was the control and “S” was experimental group in “F-vs-S” paired comparison. Each line refers to data from one gene. The color bar represents the log_10_RPKM and ranges from blue (low expression) to red (high expression)

Hierarchical clustering analysis of DEGs in F-vs-S (5,524), S-vs-T (3,468), and T-vs-FO (8714) paired comparisons identified only 164 transcripts common to all three comparisons (Fig. 3); this is presumably insufficient for regulating the conversion of several layers of ovary primordium at stage S to an ovary with developing ovules at stage FO, and implies that stage-specific transcripts are also involved.

**Fig. 4:**
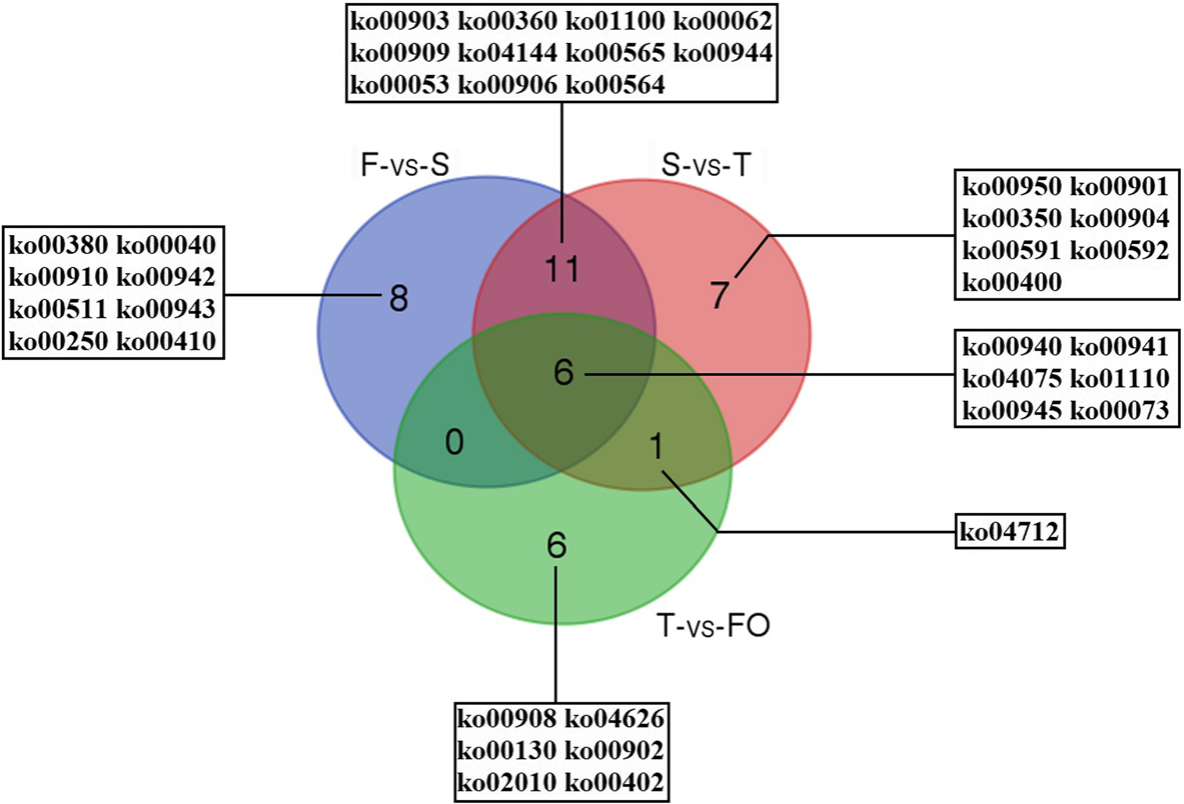
Venn diagram showing the overlaps of significantly enriched pathways in F-vs-S, S-vs-T and T-vs-FO paired comparisons

A KEGG pathway classification and functional enrichment analysis were carried out for DEGs identified in the F-vs-S, S-vs-T, and T-vs-FO paired comparisons to determine the pathways involved in the regulation of ovary differentiation. In total, 39 pathways with a Q value < 0.05 were identified: 25 pathways were significantly enriched in F-vs-S, 25 in S-vs-T, and 13 in T-vs-FO (Table 3; Additional files 6, 7 and 8: Tables S6, S7 and S8). Six significantly enriched pathways were common to the three paired comparisons (Fig. 4), including ko00940 (Phenylpropanoid biosynthesis), ko01110 (Biosynthesis of secondary metabolites), ko00941 (Flavonoid biosynthesis), ko00945 (Stilbenoid, diarylheptanoid, and gingerol biosynthesis), ko04075 (Plant hormone signal transduction), and ko00073 (Cutin, suberine, and wax biosynthesis), which are involved in the regulation of metabolism and signal transduction. These results indicate that DEGs involved in biosynthesis, metabolism, and signal transduction are important for the regulation of ovary differentiation and development in hazel.

**Table 3:** Common significant enriched pathways in F-vs-S, S-vs-T and T-vs-FO paired comparisons

KO ID	Pathway name		DEGs			Q value	
		F-vs-S	S-vs-T	T-vs-FO	F-vs-S	S-vs-T	T-vs-FO
ko00940	Phenylpropanoid biosynthesis	110 (3.3%)	109 (5.4%)	148 (2.84%)	7.20E-09	3.09E-23	1.01E-07
ko01110	Biosynthesis of secondary metabolites	513 (15.4%)	333 (16.49%)	696 (13.35%)	2.20E-13	2.01E-12	8.29E-07
ko00941	Flavonoid biosynthesis	68 (2.04%)	49 (2.43%)	82 (1.57%)	2.08E-07	1.10E-07	1.74E-04
ko00945	Stilbenoid, diarylheptanoid and gingerol biosynthesis	62 (1.86%)	52 (2.57%)	75 (1.44%)	6.82E-07	2.76E-10	3.43E-04
ko04075	Plant hormone signal transduction	235 (7.05%)	145 (7.18%)	327 (6.27%)	6.98E-06	3.30E-04	1.14E-03
ko00073	Cutin, suberine and wax biosynthesis	48 (1.44%)	37 (1.83%)	39 (0.75%)	1.12E-12	1.87E-12	2.46E-03

### DEGs in auxin biosynthesis, transport, and signal transduction pathway

We identified three unigenes encoding YUC (Indole-3-pyruvate monooxygenase) with relatively high fragments per kilobase of transcript per million mapped read (FPKM) values and significantly altered expression. In the F-vs-S paired comparisons, the three unigenes encoding *YUC* (CL11083.Contig2_All, Unigene15073_All, and CL7285.Contig3_All) were upregulated 1.62, 1.63, and 1.32-fold, respectively (Additional file 9: Table S9). The FPKM values of Unigene15073_All were the highest among the three DEGs (5.47, 18.84, 22.39, and 19.46 at stages F, S, T, and FO, respectively) (Additional file 10: Table S10), indicating high *YUC* expression levels after the first stage of development. Consistent with this observation, a DEG encoding the *auxin efflux carrier family protein PIN-FORMED* (*PIN*) (CL2654.Contig1_All) was upregulated 1.47-fold in the F-vs-S paired comparisons, with FPKM values at stages F, S, T, and FO of 8.73, 26.71, 16.78, and 20.25, respectively (Additional files 9 and 10: Tables S9 and S10). Thus, *YUC* and *PIN* were highly expressed from stage S.

Our transcriptome data revealed dozens of DEGs in the auxin signal transduction pathway (Additional file 9: Table S9). Among the five DEGs encoding *auxin influx carrier protein 1* (*AUX1*), the maximum FPKM value of Unigene23267_All at the four developmental stages was much higher than those of the other four DEGs (Additional file 10: Table S10). FPKM was maximal at stage F, and then decreased and remained at a relatively low level at subsequent developmental stages. Only one DEG encoding *TRANSPORT INHIBITOR RESPONSE 1* (*TIR1*) (CL9570.Contig1_All) was identified, and its FPKM values were 9.57, 8.93, 15.30, and 6.09 at stages F, S, T, and FO, respectively (Additional file 10: Table S10), indicating that pollination at stage S had no significant impact on expression.

AUX/IAA (auxin-responsive protein AUX/IAA) behaves as a negative regulator in auxin signaling. All three DEGs (CL1889.Contig2_All, Unigene10494_All, and CL1889.Contig1_All) encoding *AUX/IAA* were markedly downregulated in F-vs-S paired comparisons (Additional file 9: Table S9). The FMKP value of each DEG decreased and remained at a low level at later developmental stages (Additional file 10: Table S10). These results indicate that AUX/IAA may block auxin signaling at stage F and activates this pathway via a low level of expression at stages S, T and FO.

In total, 50 *ARFs* (Auxin response factors) were identified in our transcriptomic data using getorf [19] with minsize set to 150 and hmmsearch [20] with all parameters set to default according to descriptions in PlantfDB. After filtering those with low FPKM values, we obtained nine DEGs encoding *ARFs* (Additional file 9: Table S9); the FPKM value of CL8419.Contig2_All was significantly higher than that of the other eight DEGs (Additional file 9: Table S9). In F-vs-S paired comparisons, most of these DEGs were either upregulated or downregulated. The induction of auxin biosynthesis at stage S via upregulation of *YUC* may activate or repress the expression of *ARFs*.

### Auxin quantification and localization

**Fig. 5:**
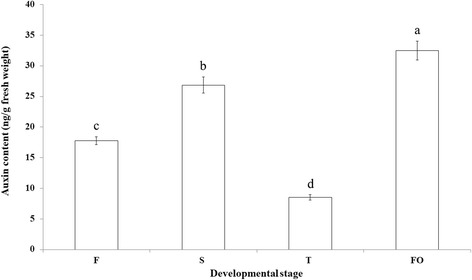
Auxin content at different developmental stages. Different small letter indicate significant difference (*P* < 0.05) by LSD test (*n* = 3)

A quantitative analysis of auxin levels at the four ovary development stages by HPLC-MS/MS revealed that levels were higher at stages S and FO than at the other two stages (*P* < 0.05) (Fig. 5), indicating that auxin biosynthesis is activated after pollination at stage S. This result was consistent with high expression of *YUC* and *PIN* starting from stage S.

**Fig. 6:**
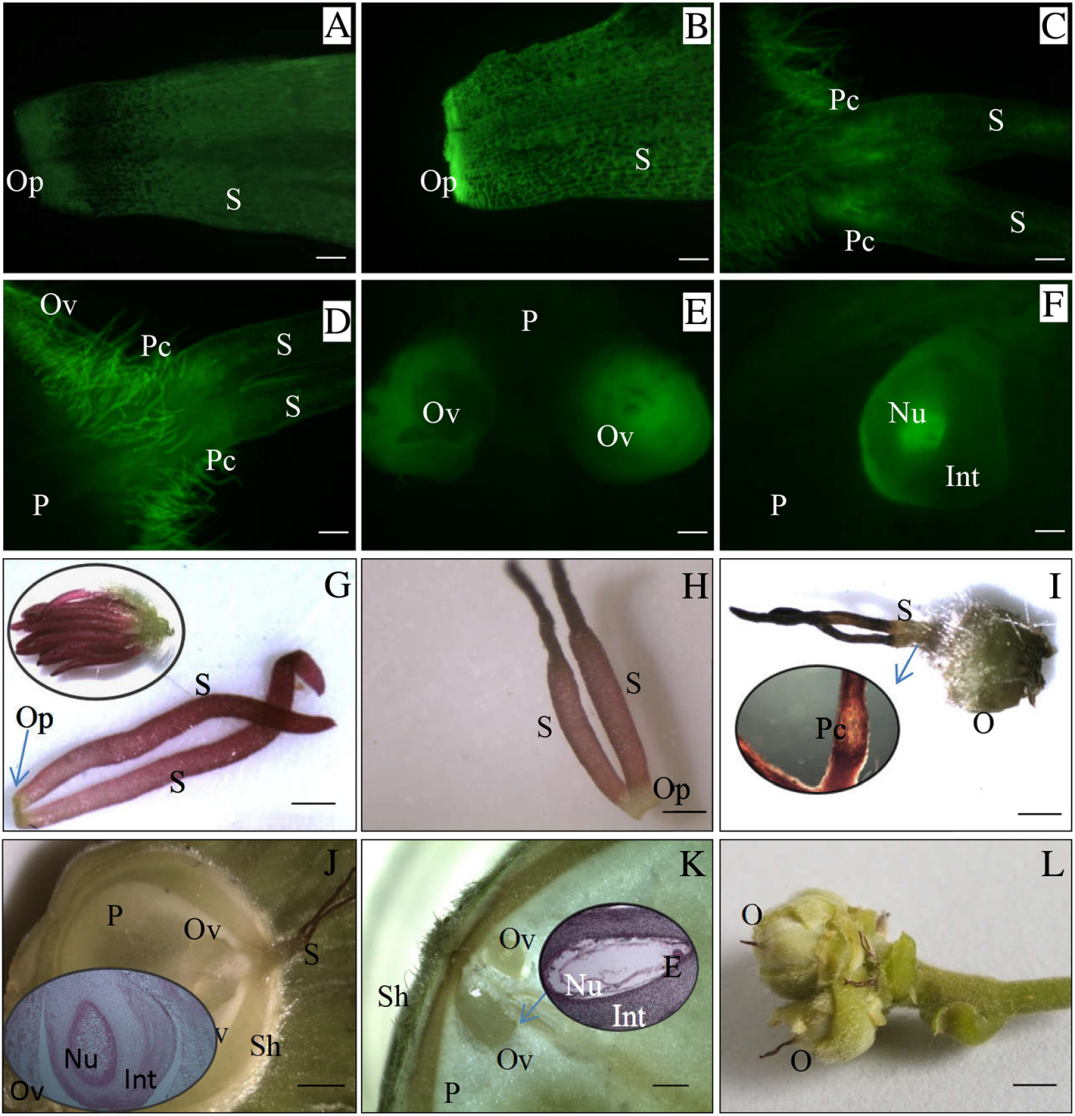
Immunohistochemical analysis of auxin distribution at different developmental stages. **a** At stage F, auxin is approximately evenly distributed throughout the stigma and early ovary primordial; the patterns of staining in female inflorescences and single female flower with two stigmas are shown in (**g**). **b** Intenser auxin staining is observed in ovary primordia at stage S; stigmas with ovary primordia are shown in (**h**). **c** and **d** Intenser auxin staining are observed in the pollen tube cavity in stigmas after rudimentary ovary formation at stage S (May 10 and May 15); an early rudimentary ovary and its pollen tube cavity are shown in (**i**). **e** Auxin staining in ovules is much higher than in other tissues of early fruit at stage T; an ovary with two ovules is shown in (**j**). **f** Auxin content in the nucellus of ovules is much higher than in other tissues of early fruit at stage FO; an ovary with ovules, and the microstructure of ovule and fruit cluster are shown in (**k**) and (**l**). Key: S, stigma; Op, ovary primordial; Pc, pollen tube cavity; O, ovary; Ov, ovule; P, parenchyma; Int, integument; Nu, nucellus; E, embryo; Sh, shell. Scale bars: A, B = 150 μm, C, D, E and F = 300 μm; G, H = 400 μm; *I* = 800 μm; J, K = 1 mm; L = 5 mm

An immunohistochemical analysis showed that auxin was uniformly distributed on the stigma and ovary primordium before pollination (Fig. 6a, g). After pollination, staining of the stigma increased compared to that at stage S. The staining for auxin was especially intense at the bottom of the stigma where primordial ovary cells were located (Fig. 6b, h). Auxin was mainly located between cell walls (Fig. 6b). The rear part of the stigma, where the cavity for the pollen tube is located, remains viable for a long period after pollination [5]. After rudimentary ovary formation at stage S, the auxin staining in the pollen tube cavity of the stigma increased, and the auxin staining of the rudimentary ovary decreased (Fig. 6c, d and i). When the ovule began to form at stage T, auxin staining was much intenser in ovules than in the parenchyma (Fig. 6e, j). During growth of the ovule at stage FO, auxin staining was much higher in the nucellus of the ovule than in the integument (Fig. 6f, k and l). Taken together, auxin is enriched at the growth center of pistillate inflorescences and young ovary after pollination.

### DEGs encoding the regulator of flower development

We identified a set of DEGs encoding quaternary proteins, including *AGAMOUS* (*AG*), *SHATTERPPOOF* (*SHP*), *APETALA* (*AP*)*1*, *SEPALLATA* (*SEP*)*1*, and *SEEDSTICK* (*STK*) (Additional file 11: Table S11). In F-vs-S paired comparisons, three DEGs encoding *AG* (CL5014.Contig1_All, CL7712.Contig2_All, and Unigene17980_All) were downregulated 2.28, 2.05, and 1.99-fold, respectively; a DEG encoding *SHP* was downregulated 2.19-fold; two DEGs encoding *SEP1* (Unigene19913_All and Unigene23003_All) were downregulated 1.35 and 3.63-fold. Thus, the expression of these FQM (floral quartet model) components was relatively high at stage F, and *AP1* (Unigene28326_All) was highly expressed at all four developmental stages (Additional file 12: Table S12).

At stage S, the FPKM values of *AP1* (Unigene28326_All) and *AG* (Unigene17980_All) were 123.38 and 29.33, respectively (Additional file 12: Table S12). At stages T and FO, ovules in pistillate inflorescences begin to differentiate and grow. Three DEGs with high FPKM values including *AP1* (Unigene28326_All), *SHP* (CL7712.Contig1_All), and *AG* (Unigene17980_All) were upregulated (Additional file 11: Table S11). Thus, the differential expression of *AP1*, *SHP*, *AG*, and *SEP* in the three paired comparisons and their relatively high FPKM values at specific developmental stages indicate that they are actively transcribed during ovary differentiation and growth.

Two DEGs encoding *NGA* (*NGATHA*) (CL1775.Contig9_All and Unigene14244_All) were identified in the F-vs-S paired comparisons (Additional file 11: Table S11); both DEGs were downregulated. Their maximum FPKM values occurred at stage F, and decreased thereafter and remained at a low level from stage S to FO (Additional file 10: Table S10).

The FPKM value of the DEG (Unigene23532_All) encoding *KAN1* (*Transcription repressor KAN1*) was maximal at stage F and then decreased and remained at a low level from stages S to FO (Additional file 10: Table S10). This is similar to the pattern observed for *NGA*. The change in FPKM value of the DEG encoding *PHB-LIKE* (Unigene26102_All) was similar to that of *KAN1*, and the value was much lower than that of KAN1 at the same developmental stage*.*

In total, five DEGs encoding *FLOWERING LOCUS* (*FL*)*C* were identified in the three paired comparisons (Additional file 11: Table S11). Among these, CL5874.Contig1_All had the highest FPKM value, and the FPKM values of all five DEGs decreased over the course of pistillate inflorescence development (Additional file 10: Table S10).

Only one DEG encoding FLOWERING LOCUS (*F*)*T* (CL10184.Contig4_All) was identified, and its FPKM value was < 2.0 at the four developmental stages (Additional file 10: Table S10), indicating that it is expressed at a low level.

Forty-five DEGs encoding *CONSTANS* or *CONSTANS-LIKE* were identified; eight remained after filtering out those with low FPKM values. Most of these DEGs had their highest FPKM value at stage FO (Additional file 10: Table S10).

Three DEGs encoding *SUPPRESSOR OF OVEREXPRESSION OF CONSTANS* (*SOC*)*1* (CL9612.Contig1_All, CL9612.Contig2_All, and Unigene18364_All) were identified in S-vs-T paired comparisons; these DEGs were downregulated 2.70, 2.90, and 5.31-fold (Additional file 11: Table S11). These results indicate that *SOC1* is more highly expressed at developmental stages F and S and regulates stigma growth and ovary primordium differentiation.

A DEG encoding *LEAFY-LIKE PROTEIN* (*LFY*) (Unigene3236_All) was downregulated 8.08-fold in the S-vs-T paired comparisons (Additional file 11: Table S11), with FPKM values of 36.48, 27.16, 0.07, and 0.07 at stages F, S, T, and FO, respectively (Additional file 10: Table S10); this pattern of variation in expression suggests a potential regulatory role at stages F and S.

Two DEGs encoding *GIGANTEA* and 12 encoding *SHORT VEGETATIVE PHASE* (*SVP)* were observed in the three paired comparisons (Additional file 11: Table S11). *GIGANTEA* expression was lowest at stage FO, and its FPKM value was much higher than that of *SVP* (Additional file 10: Table S10), indicating low abundance of *SVP*.

### Validation of DEGs by qRT-PCR

**Fig. 7:**
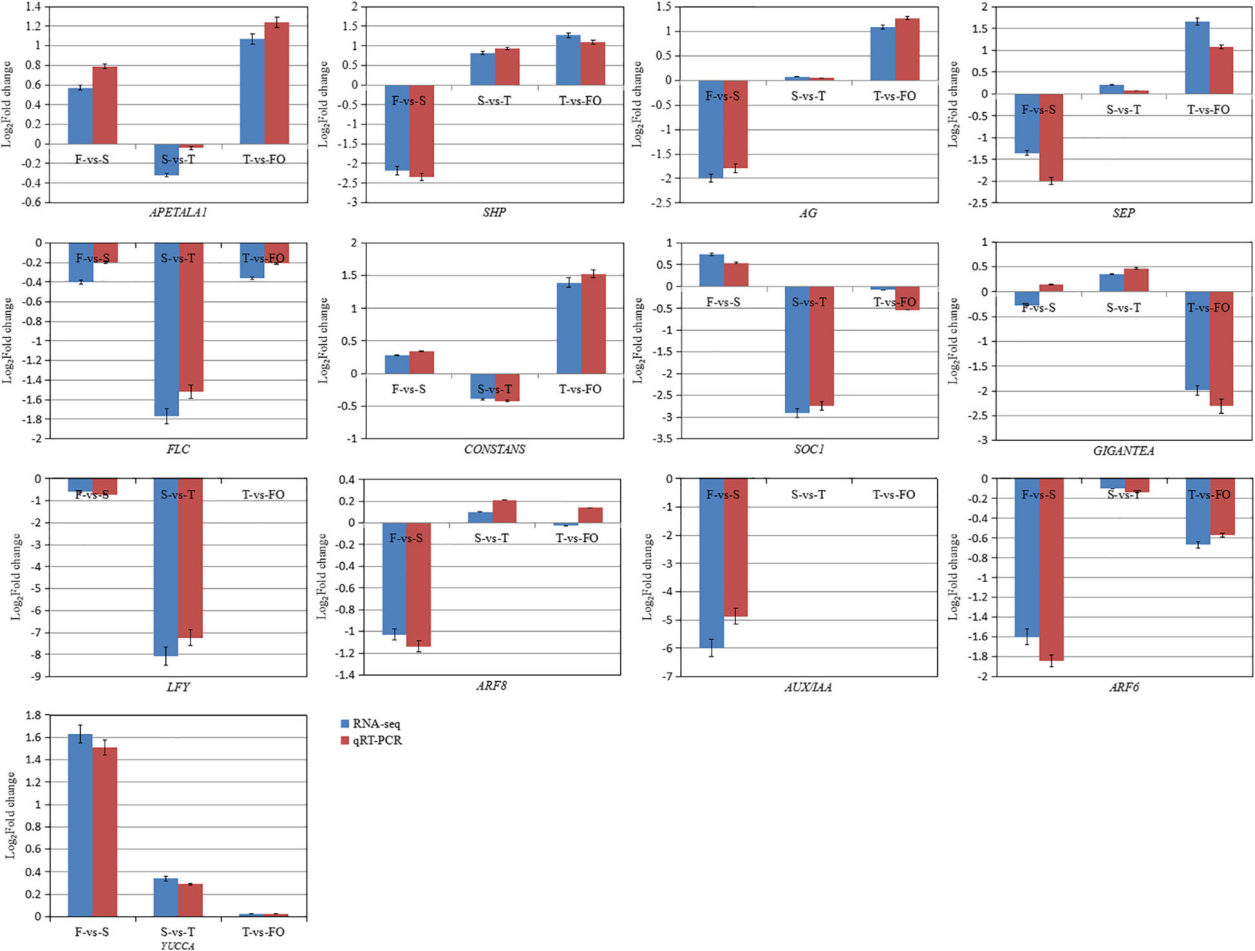
Validation of DEGs by qRT-PCR analysis. Bars represent mean ± standard deviation (n = 3)

From the DEGs identified in the F-vs-S, S-vs-T, and T-vs-FO paired comparisons, 13 genes that may be involved in the regulation of ovary differentiation and development were selected for validation by qRT-PCR analysis: *AP1*, *SHP*, *AG*, *SEP*, *FLC*, *CONSTANS*, *SOC1*, *GIGANTEA*, *LFY*, *YUC*, *AUX/IAA*, *ARF6*, and *ARF8* (Fig. 7). The expression patterns of these genes obtained in the qRT-PCR analysis were consistent with those from RNA sequencing.

## Discussion

### Auxin plays essential roles in organ patterning of pistillate inflorescences

Floral differentiation and development are complex biological processes that begin with flower bud induction and flower formation, and are regulated by environmental factors (e.g., temperature, light quality, and water) [21, 22] and endogenous signals [23]. The phytohormone auxin is critical for plant morphogenesis; its local accumulation and distribution within plant organs provide spatial information for organ patterning [24]. In reproductive tissues, auxin metabolism, intercellular transport, and intracellular signaling coordinate the development of the stamen [24–26], gynoecia [27], ovule [28], and embryo [29].

Typical gynoecia consist of four parts, namely, the stigma, style, ovary, and gynophore; these structures are arranged along an apical-basal axis. High and low auxin concentrations in the apical and basal regions, respectively, create an auxin gradient that determines tissue specification and formation [2, 30]. Accordingly, changes in the auxin gradient, for example by treatment with the auxin transport inhibitor N-1-naphthylphthalamic acid [31] or in mutants of auxin efflux (e.g., *PIN1*) [26] and auxin signaling (*ARF5/MONOPTEROS* and *ARF3*/*ETTIN*) genes [32], result in aberrant gynoecium development. The IPA pathway is the major auxin biosynthetic pathway in plants and is essential for development of both flowers and fruits. YUC family proteins catalyze the rate-limiting conversion of IPA to IAA [33]. We found here that DEGs encoding *YUC* and the auxin efflux carrier gene *PIN* were upregulated in S-vs-T paired comparisons, and that auxin content was elevated at stage S, implying active auxin biosynthesis and transport. An immunohistochemical analysis of auxin localization showed that auxin was enriched in primordial cells of the ovary. Thus, the auxin gradient in the ovary primordium is generated by the upregulation of *YUC* and *PIN*, which may be important for early ovary patterning and differentiation.

At anthesis in pistillate inflorescences, auxin was evenly distributed along the stigma. After pollination, ovary primordial cells at the bottom of the stigma accumulated auxin; this pattern of accumulation is similar to the higher auxin content in primordial leaf cells during asymmetry patterning in the gynoecium [25, 34]. The auxin influx carrier protein AUX1 is critical for polarized transport between neighboring cells and establishment of concentration gradients across plant tissues [35]. During early differentiation of the ovary primordium (stage S), *AUX1* downregulation and concomitant upregulation of *PIN* may result in the accumulation of auxin in primordial ovary cells and formation of an auxin gradient, with AUX/IAA, a negative regulator of auxin signaling, remaining low from stages S to F. Thus, auxin signaling from stage S to stage FO may promote gynoecium asymmetry patterning and ovary primordium differentiation.

*ARF6* and *ARF8* regulate the development of the gynoecium and stamen in immature flowers of Arabidopsis, including inflorescence stem elongation, anther dehiscence, gynoecium maturation, and flower bud opening [36, 37]. The *ARF6–2/ARF8–3* double mutant exhibits sterility of male and female inflorescences and produces no seeds [36]; ectopic expression *ARF6* or *ARF8* due to mutations in the microRNA miR167 causes growth arrest of ovule integuments [36]. Our transcriptome analysis revealed four DEGs encoding *ARF6* and one encoding *ARF8*. All of these were downregulated in the F-vs-S paired comparisons, indicating their high expression at stage F; their FPKM values remained at a relatively low level from stage S to stage FO. Thus, *ARF6* and *ARF8* likely play an important role in the regulation of stigma growth. However, their low levels during ovule differentiation and growth are not consistent with the sterility observed in the *ARF6–2/ARF8–3* double mutant; additional studies are needed to determine whether *ARF6* and *ARF8* are enriched in the ovule and modulate its growth.

### FQM components that regulate ovary and ovule differentiation and growth in hazelnut

The FQM suggests that tetrameric complexes of floral homeotic proteins determine floral organ identity [38]. Carpel identity is determined by a complex of two SEP and two AG proteins, while ovule identity is determined by a complex of one SEP and one AG protein along with one each of the class D proteins, SHP and/or STK. Unlike most angiosperms, stamens are absent throughout the developmental cycle of the pistillate inflorescences of hazel; in particular, stigma growth and ovary and ovule growth and differentiation are initiated in a strict temporal sequence, providing a unique model for floral development. Based on their differential expression and FPKM values, *AP1* (Unigene28326_All), *SHP* (CL7712.Contig1_All), *AG* (Unigene17980_All), and *SEP* (Unigene19913_All) were identified as the most important floral homeotic determinants of floral organ identity and are presumed to be associated with stigma identity. *AP1* and *AG* may determine early ovary differentiation after pollination at stage S, and along with SHP may regulate ovule differentiation and growth at stages T and FO. *AP1* expression was significantly enhanced after pollination at stage S, implying regulation by auxin. We also found that ARF3 is a direct target of AP2 and partly mediates AP2 function in the determination of the floral meristem [39], although further investigation is required to clarify whether *AP1* regulates ARF6 or ARF8 expression to promote crosstalk between the four homeotic proteins and the auxin signaling pathway.

Full-length *AG* (*CaMADS1*) has been cloned from pollinated styles of hazel, and northern blot and RT-PCR analyses have confirmed its role in floral organogenesis [40]. A comparison of *AG* (Unigene17980_All) and *CaMADS1* (GenBank: AF027376.1) sequences revealed 99% similarity. Furthermore, constitutive expression of *CaMADS1* in transgenic *Arabidopsis* resulted in the homeotic conversion of the first and second whorl organs [41]. Our results confirm the regulatory role of *AG* not only in floral organogenesis but also in ovule development. In particular, FPKM values of *AP1* during the four developmental stages were much higher than those of most DEGs encoding *SHP*, *AG*, *SEP*, and *STK*, highlighting the critical role of *AP1* in stigma growth, ovary differentiation, and ovule differentiation and growth. Whether *AP1* is also involved in the regulation of floral differentiation remains to be determined.

### Important regulators of flower development contribute to ovary and ovule differentiation and growth after pollination in hazelnut

*NGA* genes interact with auxin signaling components at multiple levels throughout pistil development [42, 43]. The NGA family in *Arabidopsis thaliana* comprises four members that belong to the B3-type transcription factor superfamily. In the developing apical gynoecium of *NGA* mutants, auxin synthesis is reduced via downregulation of *YUC8*, and the expression of genes involved in auxin transport and ARFs is altered throughout gynoecium development [43]. Here, we found that the maximum FPKM values of *NGA* genes occurred at stage F in the stigma of female inflorescences; however, from stage S to stage FO, FPKM values were low. These findings suggest that *NGA* genes regulate stigma growth.

PHB-LIKE proteins are important for apical meristem establishment and maintenance as well as adaxial organ identity [44], and are regulated by abaxial KAN proteins [45]. The antagonism between *KAN* and *PHB-LIKE* not only generates auxin gradients but also influences vascular development and establishment of organ asymmetry [32]. In the hazel transcriptome, *PHB* was a DEG with low abundance; this adaxial factor may be overridden by *KAN1*, which may determine the adaxial fate of the ovary primordium at stage F and provide a basis for stable ovary primordium partitioning.

*FLC* is a MADS-box gene and strong suppressor of flowering. The expression of *FLC* with low levels of DNA methylation is repressed by vernalization, resulting in early flowering [46]. In the vernalization and autonomous pathways, *FT* expression is negatively regulated by *FLC* [47]. Our results indicate that *FLC* expression decreased with pistillate inflorescence development, while the *FT* FPKM values remained at a low level throughout the four developmental stages. Vernalization is required before the anthesis of male and female inflorescences in hazel; inflorescences differentiate in the autumn and anthesis occurs in the following spring. The high level of *FLC* expression at stage F indicated the termination of vernalization. Additionally, as a strong suppressor of flowering, the low expression of *FLC* from stages S to FO implies the end of flowering suppression after pollination, which may facilitate subsequent development of pistillate inflorescences.

In order to complete their life cycle, higher plants must undergo a major developmental transition from vegetative growth to flowering. In many plants, flowering time is controlled by both environmental and endogenous signals. *CONSTANS*, which is related to GATA transcription factors, regulates the long-day pathway [48] and promotes flowering through transcriptional activation of *LFY*, a flower meristem identity gene [49]. The highest FPKM values of *CONSTANS* were at stage FO, while *LFY* was highly expressed at stages F and S. Thus, *CONSTANS* did not positively regulate *LFY*, which is contrary to previous reports [49] and underscores the complexity of floral induction. The roles of *CONSTANS* and *LFY* in the regulation of ovule growth and of stigma and early ovary primordial differentiation merit further examination.

SOC1 is a MADS-box protein that is highly expressed in reproductive organs and promotes flowering in *A. thaliana* and *Zea mays* and regulates flowering by integrating multiple signals [50–52]. *SOC1* expression levels were higher at developmental stages F and S, suggesting that *SOC1* promotes stigma growth and ovary primordium differentiation.

*GIGANTEA* induces flower differentiation in *A. thaliana* [53]. We found it to be highly expressed at stages F, S, and T, implying that it controls development of the stigma and differentiation of the ovary primordium and ovule at these developmental stages.

## Conclusions

We identified a set of genes that may regulate stigma growth, primordial ovary formation, ovule differentiation and ovule growth in hazel. At stage F, four FQM components (*AP1*, *SHP*, *AG,* and *SEP*) are highly expressed in pistillate inflorescences and which may determine floral identity and stigma growth; *FLC* and *AUX/IAA* suppress flowering, while *SOC1*, *GIGANTEA*, *LFY*, *ARF6*, *ARF8*, *NGA*, and *KAN1* are highly expressed and act as positive inducers. Thus, the balance between positive and negative regulatory factors may determine stigma growth. Similarly, *AP1*, *AG, YUC*, *PIN*, *CONSTANS*, *SOC1*, *GIGANTEA*, *LFY*, *ARF6*, *ARF8* and *FLC* may coordinate primordial ovary formation; *AP1*, *SHP*, *AG*, *CONSTANS*, *GIGANTEA*, *YUC* and *PIN* may determine ovule differentiation at stage T, and *AP1*, *SHP*, *AG*, *SEP*, *CONSTANS*, *YUC* and *PIN* may regulate ovule growth.

## Additional files

Additional file 1: **Table S1.** Primer sequences of selected unigenes in qRT-PCR. (XLSX 10 kb)

Additional file 2: **Table S2.** Categorization and abundance of reads. (XLSX 11 kb)

Additional file 3: **Table S3.** DEGs in F-vs-S paired comparisons. (XLSX 4773 kb)

Additional file 4: **Table S4.** DEGs in S-vs-T paired comparisons. (XLS 3850 kb)

Additional file 5: **Table S5.** DEGs in T-vs-FO paired comparisons. (XLS 3651 kb)

Additional file 6: **Table S6.** KEGG pathway enrichment analysis of DEGs identified in F-vs-S paired comparisons. (XLSX 23 kb)

Additional file 7: **Table S7.** KEGG pathway enrichment analysis of DEGs identified in S-vs-T paired comparisons. (XLSX 22 kb)

Additional file 8: **Table S8.** KEGG pathway enrichment analysis of DEGs identified in T-vs-FO paired comparisons. (XLSX 23 kb)

Additional file 9: **Table S9.** Selected DEGs in auxin biosynthesis, transport, and signal transduction pathway. (XLSX 12 kb)

Additional file 10: **Table S10.** FPKM values of all expressed transcripts. (XLS 7921 kb)

Additional file 11: **Table S11.** DEGs encoding regulators of flower development. (XLSX 12 kb)

Additional file 12: **Table S12.** FPKM values of DEGs encoding constituents of the floral quartet model (FQM). (XLSX 10 kb)

## Abbreviations

AG: AGAMOUS
AP: APETALA1
ARF6: Auxin response factor 6
ARF8: Auxin response factor 8
AUX: Auxin influx transporter AUX
AUX/IAA: Auxin/INDOLE-3-ACETIC ACID
FLC: FLOWERING LOCUS C
LFY: LEAFY
NGA: NGATHA
PIN: Auxin efflux carrier family protein PIN
SEP: SEPALLATA 1
SHP: SHATTERPPOOF
SOC1: SUPPRESSOR OF OVEREXPRESSION OF CONSTANS1
